# Challenges and opportunities in antimicrobial resistance research

**DOI:** 10.1099/mic.0.000999

**Published:** 2021-01-25

**Authors:** Stephen Baker

**Affiliations:** ^1^​ Cambridge Institute of Therapeutic Immunology and Infectious Disease, University of Cambridge School of Clinical Medicine, Cambridge Biomedical Campus, Cambridge, UK; ^2^​ Department of Medicine, University of Cambridge School of Clinical Medicine, Cambridge Biomedical Campus, Cambridge, UK

## Background

The United Nations Sustainable Development Goals (UN-SDGs) are 17 global goals and 169 targets adopted by all UN Member States in September 2015. These targets follow the Millennium Development Goals, but have a greater breadth of activity and are substantially more ambitious. The goals aim to tackle major global challenges by 2030 and focus heavily on inequality and climate change. As scientists aiming to understand how things work and how we can exploit these underlying mechanisms to make the world a better place, many of us will feel we have an obligation to try and better focus our work in contributing to achieving some of the major challenges outlined within the UN-SDGs. Unsurprisingly, health and the environment feature predominantly and microbiologists globally are constantly engaged in trying to address biological problems through innovative approaches and applied scientific method. The ongoing coronavirus pandemic aptly exposes the threat that infection disease poses to mankind, but also highlights how science can respond rapidly and collaboratively to generate novel methods to reduce the impact of a new infectious disease. But how do we approach such global challenges and what existing capacity do we have ready that can be directly applied to the UN-SDGs?

The COVID-19 experience has been a lesson and a severe warning shot for the challenges that humanity faces. It is increasingly apparent the virus will be with us indefinitely and there will be other pandemics in the future for which we have to respond with similar haste. But COVID-19 has shown us that we have to learn to work as a community and to make our research ever more applied to address these longer-term threats to mankind, such as pandemics, food security, environmental sustainability and antimicrobial resistance (AMR). The Microbiology Society recognizes these challenges and sees enormous potential in the microbiology research community in the UK and Ireland to jointly apply their skills in tackling major global issues. Consequently, the Society created an expansive project entitled ‘A Sustainable Future’ in 2019, which is working to define a principal role for microbiology in achieving some key aspects of the UN-SDGs. This is an ongoing mission and the scope of the UN-defined goals is exceptionally broad and often interlocked; however, engagement with the Microbiology Society’s membership identified three specific areas where the expertise of microbiologists in the UK and Ireland is particularly relevant. These areas were AMR, which is widely recognized as one of the biggest threats to humanity; the circular economy, encompassing regenerative approaches aiming to maximize the most efficient use of the world’s finite resources; and soil health, which is essential for us to sustainably feed an ever-growing global population.

## The workshop

As opposed to dictating the agenda, as a Society we have continuously sought to engage with the community as much as possible to direct ideas and to try to focus efforts on the key expertise already held within its membership. As a component of this project we held a week-long workshop in the summer of 2020 to identify challenges and opportunities for microbiological research and innovation through interdisciplinary collaboration, and interactions with industrial partners and social and political institutions in tackling AMR. As many of the concepts raised may not be feasible given current academic/industrial structures, we also asked what more could be done if there were fewer barriers, and what interventions would be needed to take the generated ideas to the next level.

Given that this workshop was performed during the ongoing pandemic, it was conducted online via focus groups chaired by recognized experts in each of the defined areas of AMR. The meeting chairs for each focus group selected two topic clusters that served to frame the discussion around challenges and opportunities for microbiology in AMR research and how we could respond as a community. The clusters for each group were: Microbiology research and innovation: (1) building a strong and resilient research community and (2) disseminating research; Interdisciplinary collaboration: (1) structures and funding and (2) managing expectations and internal communication; Industry: (1) research and industry collaboration and (2) policy, regulations and market; and Social and political institution: (1) policy context and (2) engagement and culture.

## The discussions

The focus groups were purposely composed of people from a range of backgrounds, geographical locations and career stages. The discussions were vibrant (occasionally heated) and generated a spectrum of opinions that could be directed into potential policy documents for how to both utilize and adapt the current microbiology research landscape in the UK and Ireland. Here, I outline some of the key thoughts and perceptions generated during these 90 min discussions. As a group we have tried to titrate these into some key concepts that may provide a focus into how the microbiology research community in the UK and Ireland can contribute to the global challenge of antimicrobial-resistant infections.

### Microbiology research and innovation

The microbiology research and innovation focus group agreed that we must advocate that microbiology is a major priority area within the public health system, and ensure that microbiology takes a (more) predominant position within national and international surveillance systems. Surveillance is the principal mechanism for assessing AMR trends and the way surveillance is currently structured and integrated needs to be vastly improved. Science is commonly focused on short-term individual academic achievements; therefore, longer term surveillance programmes (i.e. >5 years) are difficult to establish and maintain but we need more vision and sustainability. In Ireland, the task of setting up an efficient AMR surveillance system is likely to be directed by the EU. The UK, however, will need to develop its own strategy; this may prove more challenging out of the EU and will require the appropriate funding and direction.

There was a recognition that our knowledge exchange mechanisms are outdated and need an overhaul. Publishing is the bedrock of scientific research, but it is also a hinderance to information dissemination and career development (dependent on author position). The community needs to embrace changes in scientific publishing, have less career-incentivized approaches to the way we disseminate information, and investigate new ways of communicating findings outside of this traditional structure. Engaging with a range of different expertises will also be key; an AMR innovation and knowledge centre that pulls together capabilities from different disciplines is likely to rapidly accelerate the AMR research agenda. This could be structured as a virtual institute (i.e. such as the Alan Turing Institute) and could be championed by the Society and key influencers to act as an AMR centre of excellence for identifying and solving specific scientific problems around AMR. This type of institute could also act as a conduit for changing healthcare policy and facilitating new translational interactions with industry, which are currently poor.

Whilst the UK/Ireland microbiology research community is highly active, it lacks influence at funding organizations. Basic microbiology remains integral for our understanding and challenging of AMR, and we need to expand our knowledge of how antimicrobials work and how they behave in complex systems (i.e. systematic off-label effects and interactions with the microbiome). AMR research has historically been focused on describing and documenting, so microbiologists need to better focus their efforts on developing new and innovative solutions using routinely generated data.

### Interdisciplinary collaboration

The interdisciplinary collaboration focus group considered that the AMR community had been largely successful in bringing people together from different fields in a comparatively short time frame, suggesting there is a strong appetite to conduct interdisciplinary research around AMR. The AMR research community is fairly well established in the UK and needs to be pushed to be broader-thinking and take on increasingly ambitious projects. Basic scientific research is integral to developing new solutions, but changing behaviours, practices and engagement across society needs to be performed simultaneously. Therefore, interactions with those working in the social sciences, policy and other humanities need to be encouraged and facilitated.

AMR research is broad and habitually absorbed into different discipline-specific conferences; there are few AMR-specific conferences, most of which are focused on the molecular aspects of AMR. We as a community should advocate for a forum that brings people together from different fields and serves to communicate the value of AMR work to those who set the scientific agenda and to a wider audience. The UK/Ireland is in a strong position to lead globally on a series of AMR-focused interdisciplinary conferences to overcome barriers and provoke more cross-field research. Again, encompassing societal change alongside basic science may be a novel approach in which to form the nuclei of new collaborations and funding opportunities.

Interdisciplinary research is challenging but if researchers are to take advantage of funding calls, they must build relationships early in order to be prepared when opportunities arise. As it stands, scientific funding is again targeted towards funding individuals in the short term. There is a need for more long-term and ambitious interdisciplinary funding, providing sufficient time to establish better interdisciplinary working models and collaborations. We (as a community) can afford to be more adventurous with the projects we perform, and targeted funding is required to test innovative ideas and forge better links with industry. Innovation and knowledge centres are a potential mechanism for linking academia and industry. Such centres create a framework for future collaboration and will engage more researchers to participate in interdisciplinary research. PhD programmes in interdisciplinary research, aiming to connect academia and industry, could be a mechanism for formulating new interdisciplinary work. There is currently a national interdisciplinary PhD programme in the UK, and this mechanism is clearly a cost-effective approach to investing in work with potentially high-value returns.

### Industry

The industry focus group noted that although early-stage innovation is growing, the number of scientists trying to develop new antibiotics and diagnostics is surprisingly low. Additionally, only a limited number of the major pharmaceutical companies have active infectious disease programmes. Consequently, we need to bring industry and researchers together as much as possible to facilitate communication and develop a common set of challenges and goals for AMR research. Ensuring the two communities communicate and agree unmet needs, gaps and challenges is paramount. This arrangement needs to work both ways; the development of novel and transformative technologies is currently performed by industry, but academia is likely to contribute greatly if it becomes more involved.

Many academic researchers do not know how to interact with industry, and better engagement between both sides is required. This communication needs to highlight the way industry operates and awareness of the policy and regulatory environment. Challenge-led initiatives are very important, as they allow for industry to be involved early and for researchers to work on AMR solutions that are actually needed, they respect regulation, and they can be easily scaled. For challenge-led research to work, there must be ongoing communication throughout the process, from bench to trials.

Researchers and industry need to work with regulators to develop innovative infectious disease approaches without compromising human safety but perhaps allowing a more flexibility in trial design. It was noted that regulators in the USA and EU have established AMR advisory groups to investigate innovate approaches and how these could be accommodated. The UK regulatory landscape is overly complex and needs to be made clearer to facilitate better engagement. Significant policy reforms are happening in countries where public health research is in place, such as the WHO innovation in tuberculosis treatment in Eastern Africa. Academic funding and industrial funding need a new kind of working relationship to enable innovation, and the two groups need to renew their commitment to AMR.

There is, however, a key issue: we need significant investment in new treatments for patients infected with resistant organisms, but also need to restrict the use of new antimicrobials to mitigate resistance. Currently, this model is not viable and the antimicrobial ecosystem needs fixing. We need a mechanism around this roadblock, and combination treatments may offer a way forward. Antibacterials with antivirals can increase drug efficacy, and the combination of treatments could be an important transformative tool. Combining older technologies, such as antimicrobial classes that are no longer used, with novel innovations, such as monoclonal antibodies, may provide revolutionary new approaches in infectious disease therapeutics.

### Social and political institutions

Given the time at which the workshop was conducted, the social and political institutions group initially discussed how COVID-19 had the potential to positively and negatively impact AMR research. Overall, it was considered that the impact of COVID-19 on AMR had, in the short term, been negative, with many projects stopping, people being unable to work in laboratories and funding being diverted into tackling the pandemic. UK GDP has fallen, and consequently the amount of funding available as Official Development Assistance is likely to be substantially reduced in coming years, which may impact international AMR programmes. Alternatively, COVID-19 will inevitably have knock-on effects with respect to AMR from the increased use of antibiotics in COVID-19 patients. Additionally, there is now a receptive general audience for the impact of infectious disease on human populations. Whether there is an increased risk of generating AMR as a result of COVID-19 remains to be demonstrated. However, it appears that the number of secondary infections in COVID-19 patients may have increased antibiotic usage in hospitals.

More generally, the panel agreed that a better understanding of the ‘one health’ approach and the environmental dimension of AMR between humans and animals was needed, rather than a focus on environmental health or the impact that the environment has on human health. Stewardship programmes that promote improved prescribing behaviours have been successful over time with significant reductions in antimicrobial usage in livestock; lessons from small-scale animal health studies could be applied globally and adapted into human health care. Again, enhanced surveillance was outlined as a necessity, with adaptive culture-independent methods being implemented for well-defined programmes.

There was a general agreement that the microbiology community could do more to educate the public about AMR and how research is being used to tackle it. Microbiology could be taught in schools and become a component of the secondary school syllabus. Through education the profile of AMR could be raised and stressed that it is an urgent threat to human health and not a hypothetical problem. AMR may not yet be being taken seriously and there is a lack of recognition of how AMR contributes to patient death. A potential solution would be including AMR-related infection on death certificates as a contributing factor. A further possibility would be the labelling of antibiotics with information of how these drugs affect the patient’s microbiome and increase the personal and population risk of drug-resistant infections.

## Summary

The current landscape of research into AMR in UK/Ireland is broad and highly active, and it is an arena in which we can have a major global impact. However, the challenge that confronts us is significant and we need to augment some aspects of our current AMR research agenda to provide new solutions to AMR infections and to deliver the UN-SDGs. The information and the issues presented here were identified and discussed by leading experts. These opinions are not exhaustive, but do highlight some of the major issues of the current academic structure and outline some potential solutions for how we move forward. We need to think beyond publication and aim to create more sustainability in our research, with better rewards for the truly innovative. Whilst basic research is fundamental to understanding the how and why, we also need to be more applied, aiming to translate our findings into tangible new interventions through better interactions with other disciplines and industrial partners. Lastly, AMR is going to be an enduring problem and we need longer term support to understand and overcome it; the creation of an AMR innovation and knowledge centre could provide this platform and become the hub of new innovation, interdisciplinary research, academic/industry links and policy. AMR is one of the biggest healthcare challenges we currently face and we as a Society hope our members can rise to the challenge.

